# Preface

**DOI:** 10.1080/15438600490424334

**Published:** 2004


*Experimental Diab. Res.*, 5:1, 2004
Copyright © Taylor and Francis Inc.
ISSN: 1543-8600 print / 1543-8619 online
DOI: 10.1080/15438600490424334

### Preface

In the last number of years, there has been a renewed interest in the physiological function of proinsulin C-peptide and the effects of its substitution on type 1 diabetic complications.

It was originally believed that the only biological function of C-peptide was that of a spacer in the biosynthesis of the insulin molecule and that it was then cleaved off from insulin without any further biological effect. However, in recent years, several physiological effects of C-peptide have been established, whose perturbations in C-peptide–deficient type 1 diabetes play important roles in the pathogenesis of several chronic complications, such as nephropathy, retinopathy, neuropathy, and primary encephalopathy.

So, for instance, there is overwhelming evidence establishing the effect of C-peptide on $Na^+$,$K^+$-ATPase and NO activities in several target tissues and on gene regulatory effects of various trophic factors and their receptors, including the insulin receptor. Furthermore, it has modulatory post-translational effects on adhesive molecules determining protein-protein interactions. Recently, it was also shown that C-peptide has gene regulatory effects on extracellular matrix proteins and that it exerts antiapoptotic effects.

Despite the evolving wealth of information regarding its pathophysiological place in type 1 diabetes, the receptor through which it exerts its activities remains elusive. Nevertheless, it has been shown that C-peptide binds to cell membranes with high affinity, and that it shares with insulin many of its signaling characteristics. Limited clinical investigations seem to confirm much of the experimental data with respect to the more severe microvascular complications associated with type 1 diabetes. These include beneficial effects of C-peptide replacement on somatic and autonomic neuropathy, nephropathy, retinopathy, and the lately recognized primary encephalopathy.

This special issue of *Experimental Diabesity Research* presents a compilation of up-to-date reviews by the leaders in C-peptide research. I am hopeful that this issue will be a valuable comprehensive update on C-peptide research that will benefit clinicians and diabetologists caring for the increasing number of type 1 patients, as well as for clinical and basic investigators and students with an interest in the research into diabetes and its devastating complications.

*Anders A. F. Sima*
*Detroit, December, 2003*

